# Genetic clues to COVID-19 severity: exploring the stromal cell-derived factor-1/CXCL12 rs2839693 polymorphism in adult Egyptians

**DOI:** 10.1186/s12879-023-08691-1

**Published:** 2023-10-19

**Authors:** Osama H. Korayem, Amr E. Ahmed, Mohamed H. Meabed, Doaa M. Magdy, Wafaa M. Abdelghany

**Affiliations:** 1 Biotechnology and Life Sciences Department, Faculty of Postgraduate Studies for Advanced Sciences, Beni-Suef University, Beni-Suef, Egypt; 2 Department of Pediatrics,Faculty of Medicine, Beni-Suef University, Beni-Suef, Egypt; 3 Department of Chest Disease and Tuberculosis, Faculty of Medicine, Assiut University, Assiut, Egypt; 4 Department of Clinical and Chemical Pathology, Faculty of Medicine, Cairo University, Cairo, Egypt

**Keywords:** CXCL12, COVID-19, Mild, SARS-CoV-2, Polymorphism, Severe

## Abstract

**Background:**

A novel corona virus called SARS-CoV-2 was identified at the end of December 2019, and the illness induced by it was designated as coronavirus disease 2019 (COVID-19). Severity of the disease could vary significantly since most of the infected individuals experience mild to moderate respiratory symptoms and recover without specialized care. Genetic polymorphisms have implications in influencing the varying degrees of COVID-19 severity. This study aims to assess the potential association between the CXCL12 rs2839693 polymorphism and the severity of COVID-19 in Assiut University Quarantine Hospital during the period from May 2022 to August 2022.

**Methods:**

The present study is a cross-sectional study and is applied to 300 COVID-19 patients confirmed by RT-PCR admitted to Assiut University Quarantine Hospital from May 2022 to August 2022. Based on the clinical symptoms, the recruited participants had been divided into two groups. Group I involved mild or moderate cases; Group II involved severe or critical conditions. The rs2839693 polymorphism was detected by real time PCR using TaqMan assay probe.

**Results:**

The frequency of the T allele and the TT genotype was significantly higher in the severe or critical group compared with the mild or moderate group (*p* value < 0.001). C-reactive protein (CRP) and D-dimers are significantly elevated in the combined variants (CT + TT) and the TT compared with the CC (*P* value 0.006 and 0.017 respectively) and the CC,CT genotypes (*p* value 0.019 and 0.002 respectively). The combined variants (CT + TT) of CXCL12 were found to be independent predictors to severe or critical COVID-19 risk with *P* value =  < 0.001, OR = 3.034& 95% CI = 1.805–5.098.

**Conclusion:**

Our findings revealed that CXCL12 rs2839693 had a role in the development and seriousness of COVID-19. Patients with the TT genotype or the T allele at increased risk developed severe or critical rather than mild or moderate disease.

## Introduction

A novel corona virus called severe acute respiratory syndrome coronavirus 2 (SARS-CoV-2) was identified at the end of December 2019, and the illness induced by it was designated as coronavirus disease 2019 (COVID-19) [1].

Most patients infected with SARS-CoV-2 do not exhibit any symptoms, yet occasionally mild or moderate symptoms may appear [2].

Genetic variations in SARS-CoV-2 may develop mutant forms that are distinct from the original strains. Only just few variants in SARS-CoV-2 were considered as "variants of concern" (VOCs) by the World health Organization (WHO). According to the WHO's most current epidemiological report, five SARS-CoV-2 VOCs (Alpha, Beta, Gamma, Delta, and Omicron) have just been detected since the epidemic began on December 11, 2019 [3].

Under the guidance of the WHO, Egypt has developed a persuasive care strategy to address the COVID-19 pandemic. The country's comparatively lower infection rates, including factors like high temperatures, high levels of humidity, early Bacille Calmette-Guérin (BCG) vaccine usage, may be a distinct virus strain. In addition, it describes the situation and the preventative steps the nation has made to deal with the pandemic [4].

Vulnerability to SARS-CoV-2, depends on many factors such as genetic polymorphisms which studied in the following: transmembrane protease serine 2 (TMPRSS2), tumor necrosis factor-alpha (TNF-α), interferon-gamma (IFN-γ), the ABO blood type, and angiotensin-converting enzyme II (ACE2) [5].

Chemokines, belonging to cytokine superfamily, are proteins with low-molecular-weight. They stimulate the movement of immune cell by attaching to immune cells surface receptors. Chemokines can be categorized into four subfamilies: CC, CXC, XC, and CX3C [6].

Numerous chemokines are involved in various viral diseases including hepatitis B virus, influenza virus, human immunodeficiency virus, respiratory syncytial virus, and hepatitis C virus. Besides, coronaviruses comprises SARS-CoV-2 [7].

Chemokine synthesis during viral infection is a crucial step in guiding immune cells to the infection site where the virus is present. However, excessive immune cell targeting results in severe inflammation trigger the acute respiratory distress syndrome (ARDS), a frequent COVID-19 consequence. Chemokine control is a crucial part in managing viruses. Increasing understanding of the chemokine profile in COVID-19 could improve our knowledge of the immune-pathological pathways of SARS-CoV-2 infection [8].

The CXCL12, also known as stromal cell-derived factor-1 (SDF-1), rs2839693 variant is reported in many diseases including HIV-1 [9], Pulmonary tuberculosis [10], breast cancer [11], and coronary artery disease [12].

A variety of immune cells, fibroblasts, and epithelial cells produce CXCL12 are found on human chromosome 10q11.1 [11]. As opposed to the exon that codes for the functional protein, the rs2839693 SNPs are intron_variants [13].

This study aimed to assess the potential association between the CXCL12 rs2839693 polymorphism and the severity of COVID-19 in Assiut University Quarantine Hospital in the period from May 2022 to August 2022.

## Materials and methods

### Study design and participants

Our current research is a cross-sectional investigation administered on 300 adult Egyptian patients diagnosed with COVID-19. These patients incorporated to Assiut University Quarantine Hospital outpatient clinics, inpatient departments and intensive care units (ICU) during the period from May 2022 to August 2022.The diagnosis of COVID-19 depended on reverse transcription polymerase chain reaction (RT-PCR) of nasal or pharyngeal swabs positive result, according to the guidelines of WHO [14]. The participants were subjected to a full history taking, appropriate clinical examination and laboratory investigations which included complete blood count, arterial blood gases, ferritin, C-reactive protein (CRP) and D-dimer. The incorporated patients were classified into 2 groups depending on clinical signs: group I with mild or moderate individuals; Group II with severe or critical individuals [15]. Patients who encountered exclusion criteria were isolated from our study: HIV-1 2, Systemic lupus erythematosus, autoimmune disease, Celiac disease, Cancer, Neurological disorders, chronic obstructive pulmonary disease (COPD), and Asthma.

### Study variables

The onset time was defined as the day on which individuals developed any symptoms. The degree of COVID-19 severity was estimated by using the guidelines of WHO for SARS-CoV-2 diagnosis and treatment. Mild or moderate patients without dyspnea showed SpO2 ≥ 94%. The presence of one or more of the following characteristics was considered as a severe or critical case: (a) a SpO2 of less than 93% at rest; (b) a respiratory rate of more than 30 breaths per minute; and (c) an oxygenation index of less than 300 mm Hg (artery partial pressure of oxygen/inspired oxygen fraction, PaO2/FiO2) [15].

All individuals underwent chest computed tomography (CT) scans since pulmonary lesions cannot be ruled out by a normal chest X-ray, particularly in patients with no symptoms and moderate instances.

### Sample size calculation

According to Takazawa & Morita, 2020 the sample size could be calculated from the following equation [16].

$$\text{n}=\left\{\left(\frac{\mathrm{Za}/2+{\mathrm Z}_{\mathrm B}}{{\mathrm P}_1-{\mathrm P}_2}\right)\right\}^2\left(\mathrm p1\mathrm q1+\mathrm p2\mathrm q2\right)$$

*n* = sample size, Z a/2 (The critical value that divides the central 95% of the Z distribution), ZB (The critical value that divides the central 20% of the Z distribution), p1 = Level in group I, p2 = Level in group II, q = 1-p.

This study is based on the work of Belperio et al., 2004. The sample size was calculated using Epi Info STATCALC using the following assumptions:—95% two-sided confidence level, with an 80% power. With a 5% margin of error, the odds ratio estimated was 1.115. The ultimate sample size calculated from the Epi- Info output was 140 [17].

### Specimen collection

Two ml of venous blood were obtained from all COVID-19 participants by a sterile venipuncture in a vacutainer tube containing EDTA as an anticoagulant for real-time PCR investigation of rs2839693 polymorphism in the CXCL12 gene; sample was kept frozen at -20 °C until DNA extraction.

### Genotyping assay

#### DNA extraction

Patients with COVID-19 had their genomic DNA extracted from EDTA peripheral blood by using the Genomic DNA Purification Kit supplied by Thermo Fisher Scientific according to Whole Blood Genomic DNA Purification Main Protocol. Catalog number: K0512.

#### Genotyping of CXCL12 rs2839693

DNA was amplified using the Genotyping TaqPath 1-Step Multiplex Master Mix from Thermo Fisher (Cat. No. A28521). The steps in the amplification process were as follows: Enzyme activation for 10 min at 95 degrees, followed by 35 cycles of denaturation for 15 s at 95 degrees, annealing for 1 min at 60 degrees, and elongation for 1 min at 72 degrees. The context sequence [VIC/FAM]: GAAGGGGACGACAGGATGCTCTAGG[C/T]ACCTGGGGAGGGGAGAATGGAGAGC was utilised using the TaqMan ready-made SNP assay (Thermo Fisher; Catalogue no. 4351379). A 20 μL PCR mixture was comprised of 3μL extracted DNA, 0.5μL SNP assay, 5μL Master Mix, and 11.5μLdistilled water was carried out. The Applied Biosystems 7500 real-time polymerase chain reaction (real time PCR) apparatus was used.

### Statistical analyses

With the use of the IBM SPSS software package version 20.0, data were inputted into the computer and analyzed [18]. Numbers and percentage were used to describe qualitative data. The normality of the distribution was examined using the Kolmogorov–Smirnov test. Utilizing range (minimum and maximum), mean, and standard deviation, quantitative data were reported. At the 5% level, significance of the results was determined. The first test, the Chi-square test, was employed to compare various groups for categorical variables. The second test, Student t-test, was employed to compare two examined groups for typical quantitative variables. The third test, Mann Whitney test, was employed to compare two investigated groups in order to compare unusual quantitative variables. The risk factors for clinical course in patients were determined using multivariable logistic regression analysis and presented as odds ratios and 95% Cis.

## Results

### Demographic and clinical characteristics of COVID-19 patients

Table 1 shows the demographic and clinical features of COVID-19 patients. In this cross-sectional study, we included 300 COVID-19 patients. Their ages ranged between 36 and 85 years, with a median age of 66 years. The patients' gender ratio was 56.3% male and 43.7% female. Most of patients were non-smokers and vaccinated. Hypertension was the most frequent comorbid disease while fatigue and dry cough were the most frequent symptom.

**Table 1:** Demographic and clinical characteristics of COVID-19 patients

	**No**	**(%)**
**Age (years)**		
≤ 65	144	48.0
> 65	156	52.0
Median (Range)	66.00 (36–85)	
**Sex**		
Male	169	56.3
Female	131	43.7
**Smoking**		
No	182	60.7
Yes	118	39.3
**Vaccination(AstraZeneca or *Pfizer*)**		
No	92	30.7
Yes	208	69.3
**Co-morbid diseases**		
None	91	30.3
Hypertension	107	35.7
Liver disease	17	5.7
Renal disease	39	13.0
DM	78	26.0
Thyroid disease	12	4.0
Heart disease	13	4.3
**Clinical course**		
Mild or moderate illness	156	52.0
Severe or critical illness	144	48.0
**Symptoms**		
Fever	73	24.3
Sore throat	110	36.7
Dry cough	161	53.7
Headache	69	23.0
Dyspnea	144	48.0
Diarrhea	21	7.0
Myalgia	123	41.0
Fatigue	165	55.0
Nausea	44	4.7
Vomiting	15	5.0
Anosmia	85	28.3
Gustatory dysfunction	69	23.0
Dysarthria	9	3.0

*DM* Diabetes Mellitus, *No* number, *%* percentage

### Physical examination, laboratory evaluation, and CT findings of COVID-19 patients

Our data showed that lymphocyte count in most cases was within the normal values, while lymphopenia was detected in 84 (28.0%) of COVID-19 patients. Ferritin, cell reactive protein (CRP), and D-dimers were elevated in most cases. Regarding CT findings, our results revealed that bilateral ground glass opacity (GGO) was the most frequent CT abnormality (Table 2).

**Table 2:** Distribution of COVID-19 patients according to physical examination, laboratory investigation and CT findings on admission

Physical examination findings on admission	No	(%)
**Temperatures (°C)**		
< 38.0	206	68.7
≥ 38.0	94	31.3
Median (Range)	38.00 (36.2–40)	
**Laboratory investigation**		
**lymphocyte count (× 10**^**9**^**/L)**		
< 1.0	84	28.0
1.0–4.0	185	61.7
> 4.0	31	10.3
Median(Range)	1.40 (0.4–5.8)	
**Leucocyte (× 10**^**9**^**/L)**		
< 4.0	61	20.3
4.0–10.0	142	47.3
> 10.0	97	32.4
Median(Range)	7.70 (1.8–16.2)	
**Platelets × 10**^**3**^**/mL**		
≤ 100	44	14.7
> 100	256	85.3
Median(Range)	244.00 (71–420)	
**CRP level (mg/L)**		
≤ 5	50	16.7
> 5	250	83.3
Median(Range)	48.00 (4–768)	
**Ferritin level (μg/L)**		
≤ 300	100	33.3
> 300	200	66.7
Median(Range)	404.50 (10–2500)	
**D-dimers level (μg/L)**		
< 0.5	120	40.0
0.5–1.0	92	30.7
> 1.0	88	29.3
Median(Range)	0.70 (0.1–3.3)	
**Imaging**		
**CT findings**		
Normal	27	9.0
Bilateral GGO	205	68.3
Pneumonic consolidation	107	35.7

*C* degree Celsius, *CRP* C-reactive protein, *CT* Computed Tomography, *GGO* Ground-glass opacity, *L* liter, *mL* millileter, *mg/L* milligram/Liter, *μg/L* microgram/Liter, *No* number, *%* percentage

### Treatment and outcomes of COVID-19 patients

According to Table 3, most patients received antibiotic and antiviral treatment. A large proportion of patients did not need oxygen therapy while, mask oxygen was needed in half of the patients. Outcomes showed that the percentage of patients who needed home management was equal to those needed hospitalization without intensive care unit (ICU); while around half of the patients admitted to ICU, and 39 (13.0%) patients died. Duration of ICU stay ranged between 1.0–13.0 days with a median value 7.0 days. The median duration of in-hospital stay was 7.0 days and a range (1.0–15.0) days. Duration of recovery ranged between 2.0–48.0 days with median value 15.0 days.

**Table 3:** Distribution of COVID-19 patients according to treatment and outcomes

Treatment modalities	No	(%)
Antibiotic	288	96.0
Antifungal	18	6.0
Antiviral	232	77.3
Glucocorticoids	176	58.7
Clexane	92	30.7
**Oxygen therapy**		
None	73	24.3
Nasal cannula	78	26.0
Mask oxygen	150	50.0
Non-invasive mechanical ventilation	64	21.3
Invasive mechanical ventilation	18	6.0
**Outcomes**		
Home management	78	26.0
Hospitalization without ICU	78	26.0
ICU	144	48.0
Death	39	13.0
**Duration of ICU stay (days)**		
Median(Range)	7.00 (1.0–13.0)	
**Duration of in-hospital stay (days)**		
Median(Range)	7.00 (1.0–15.0)	
**Duration of recovery (days)**		
Median(Range)	15.00 (2.0–48.0)	

*ICU* Intensive care unit. *No* number, *%* percentage

### Genetic findings of CXCL12 rs2839693 for COVID-19 patients

Table 4 shows a highly statistical significant difference between mild or moderate and severe or critical groups regarding allelic and genotyping frequencies (*p* value < 0.001). The frequency of the TT genotype and the T allele were higher in the severe or critical group than in the mild or moderate group. Regarding genotypes, patients with the CT + TT genotype had 3.08 higher risks to develop severe or critical COVID-19 than the CC genotype. Regarding alleles, patients with the T allele had 2.91 higher risks to develop severe or critical COVID-19 than the C allele.

**Table 4:** Comparison according to genetic (CXCL12 rs2839693) findings of COVID-19 patients

**CXCL12 rs2839693**	**Mild or moderate (*n* = 156)**		**Severe or critical (*n* = 144)**		**OR (95%CI)**	*P* value*
	**No**	**%**	**No**	**%**		
**Genotypes**						
CC	126	80.8	83	57.6	0.32(0.19–0.54)	& < 0.001*
CT	30	19.2	54	37.5	2.52(1.50–4.25)	& < 0.001*
TT	0	0.0	7	4.9	0.47(0.41–0.53)	&0.005*
CT + TT	30	19.2	61	42.4	3.08(1.84–5.18)	& < 0.001*
**Alleles**						
C	282	90.4	220	76.4	2.91(1.83–4.62)	& < 0.001*
T	30	9.6	68	23.6		

^&^Fisher’s Exact test. *p* values represent statistical significance < 0.05

^*^Significant. *OR* Odds ratio. *CI* confidence interval

Multivariate logistic regression analysis showed that the combined variants (CT + TT) of CXCL12 were found to be independent predictors to severe or critical COVID-19 risk with *P* value =  < 0.001, OR = 3.034& 95% CI = 1.805–5.098 (Table 5).

**Table 5:** Multivariate logistic regression of genotypes against clinical COVID19 stage

		*P* value^*^	**OR**	**95% C.I**	
				**Lower**	**Upper**
**Severe or critical COVID19**	**Genotyping of CXCL12** CC/CT + TT	< 0.001^*^	3.034	1.805	5.098

*p* values represent statistical significance < 0.05

^*^Significant. *OR* Odds ratio. *CI* confidence interval

### Patient demographic data, comorbidity, and symptoms according to CXCL12 rs2839693 genotypes

Subsequently, we compared demographic data, comorbidity, and symptoms between CXCL12 genotypes (CC vs. CT + TT and CC vs. CT vs. TT) as presented in Table 6. Significant associations of genotypic distributions with dyspnea, vomiting, and dysarthria of COVID-19 patients were detected (*P* value < 0.001, 0.015, and < 0.001 respectively). Dyspnea was more prevalent in the combined CT + TT and the TT genotypes compared with the CC and the CT, CC genotypes, respectively. Vomiting and Dysarthria were significantly prevalent in the TT genotype compared with the CT, CC genotypes. Regarding clinical course of the disease, we found that severe or critical illness was associated with the combined CT + TT and the TT genotypes where it was more prevalent in the combined CT + TT, and the TT genotypes was compared with the CC and the CT, CC genotypes, respectively.

**Table 6:** Comparison between genotypes (CXCL12 rs2839693) according to demographic data, comorbidity, and symptoms

	**CXCL12 rs2839693**							
	**CC** **(*n* = 209)**	**CT + TT** **(*n* = 91)**	**OR (95%CI)**	*P* value*	**CC** **(*n* = 209)**	**CT** **(*n* = 84)**	**TT** **(*n* = 7)**	*P* value*
	**No. (%)**	**No. (%)**			**No. (%)**	**No. (%)**	**No. (%)**	
**Age (years)**								
≤ 65	101(48.3)	43(47.3)	1.04 (0.64–1.71)	&0.900	101(48.3)	42(50.0)	1(14.3)	&0.189
> 65	108(51.7)	48(52.7)			108(51.7)	42(50.0)	6(85.7)	
Median (Range)	66 (36–93)	70 (47–82)	------	§0.221	66 (36–93)	70 (47–82)	62 (58–68)	§0.042*
**Sex**								
Male	113(54.1)	56(61.5)	0.74 (0.45–1.22)	&0.256	113(54.1)	52(61.9)	4(57.1)	&0.473
Female	96(45.9)	35(38.5)			96(45.9)	32(38.1)	3(42.9)	
**Smoking**								
No	131(62.7)	51(56.0)	1.32 (0.80–2.17)	&0.305	131(62.7)	47(56.0)	4 (57.1)	&0.556
Yes	78(37.3)	40(44.0)			78(37.3)	37(44.0)	3(42.9)	
**Vaccination(AstraZeneca or *Pfizer*)**								
No	62(29.7)	30(33.0)	0.86 (0.51–1.46)	&0.588	62(29.7)	27(32.1)	3(2.9)	&0.714
Yes	147(70.3)	61(67.0)			147(70.3)	57 (67.9)	4(57.1)	
**Co-morbid diseases**								
None	69(33.0)	22(24.2)	0.65 (0.37–1.13)	&0.135	69(33.0)	22(26.2)	0(0)	&0.109
Hypertension	76(36.4)	31(34.1)	0.90 (0.54–1.52)	&0.793	76(36.4)	29(34.5)	2 (28.6)	&0.884
Liver disease	10(4.8)	7(7.7)	1.66 (0.61–4.50)	&0.415	10(4.8)	6(7.1)	1(14.3)	&0.445
Renal disease	23(11.0)	16(17.6)	1.73 (0.86–3.45)	&0.136	23(11.0)	14(16.7)	2(28.6)	&0.198
DM	52(24.9)	26(28.6)	1.21 (0.70–2.10)	&0.567	52(24.9)	23(27.4)	3(42.9)	&0.534
Thyroid disease	7(3.3)	5(5.5)	1.68 (0.52–5.43)	&0.522	7(3.3)	5(6.0)	0(0)	&0.508
Heart disease	8(3.8)	5(5.5)	1.46 (0.47–4.59)	&0.543	8(3.8)	5(6.0)	0(0)	&0.514
**Symptoms**								
Fever	49(23.4)	24(26.4)	1.17 (0.66–2.06)	&0.661	49(23.4)	20(23.8)	4(57.1)	&0.123
Sore throat	82(39.2)	28(30.8)	0.69 (0.41–1.16)	&0.193	82(39.2)	26(31.0)	2(28.6)	&0.373
Dry cough	116(55.5)	45(49.5)	0.78 (0.48–1.28)	&0.378	116(55.5)	42(50.0)	3(42.9)	&0.587
Headache	46(22.0)	23(25.3)	1.19 (0.67–2.13)	&0.553	46(22.0)	21(25.0)	2(28.6)	&0.807
Dyspnea	83(39.7)	61(67.0)	3.09 (1.84–5.18)	& < 0.001*	83(39.7)	54(64.3)	7(100)	& < 0.001*
Diarrhea	17(8.1)	4(4.4)	0.52 (0.17–1.59)	&0.327	17(8.1)	3(3.6)	1(14.3)	&0.286
Myalgia	82(39.2)	41(45.1)	1.27 (0.77–2.09)	&0.373	82(39.2)	39(46.4)	2(28.6)	&0.419
Fatigue	117(56.0)	48(52.7)	0.88 (0.54–1.44)	&0.616	117(56.0)	45(53.6)	3(42.9)	&0.753
Nausea	31(14.8)	13(14.3)	0.96 (0.48–1.93)	&1.000	31(14.8)	11(13.1)	2(28.6)	&0.535
Vomiting	9(4.3)	6(6.6)	1.57 (0.54–4.54)	&0.399	9(4.3)	4(4.8)	2(28.6)	&0.015*
Anosmia	63(30.1)	22(24.2)	0.74 (0.42–1.30)	&0.331	63(30.1)	21(25.0)	1(14.3)	&0.478
Gustatory dysfunction	53(25.4)	16(17.6)	0.63 (0.34–1.17)	&0.179	53(25.4)	15(17.9)	1(14.3)	&0.331
Dysarthria	4(1.9)	5(5.5)	2.98 (0.78–11.4)	&0.136	4(1.9)	3(3.6)	2(28.6)	& < 0.001*
**Clinical course**								
Mild or moderate illness	126(60.3)	30(33.0)	3.09 (1.84–5.18)	& < 0.001*	126(60.3)	30(35.7)	0(0)	& < 0.001*
Severe or critical illness	83(39.7)	61(67.0)			83(39.7)	54(64.3)	7(100)	

^§^Mann Whitney test. &Fisher's Exact test. *P* values represent statistical significance < 0.05

^*^Significant. *OR* Odds ratio. *CI* confidence interval. *DM* Diabetes Mellitus

### Laboratory evaluation and CT findings according to CXCL12 rs2839693 genotypes

Table 7 shows statistical significant differences between genotypes, regarding CRP, D-dimers, and ferritin. Where CRP and D-dimers were significantly elevated in the combined CT + TT, and the TT was compared with the CC (*P* value 0.006 and 0.017 respectively) and the CC, CT genotypes (*p* value 0.019 and 0.002 respectively); while Ferritin was significantly elevated in the TT compared with the CC, CT genotypes (*P* value 0.050).

**Table 7:** Comparison between genotypes (CXCL12 rs2839693) according to Laboratory investigation and CT findings

	**CXCL12 rs2839693**							
	**CC** **(*n* = 209)**	**CT + TT** **(*n* = 91)**	**OR (95%CI)**	*P* value*	**CC** **(*n* = 209)**	**CT** **(*n* = 84)**	**TT** **(*n* = 7)**	*P* value*
	**No. (%)**	**No. (%)**			**No.(%)**	**No. (%)**	**No. (%)**	
**Laboratory investigation**								
**lymphocyte count (× 10**^**9**^**/L)**								
< 1.0	62(29.7)	22(24.2)	------	#0.579	62(29.7)	21(25.0)	1(14.3)	#0.635
1.0–4.0	125(59.8)	60(65.9)			125(59.8)	54(64.3)	6(85.7)	
> 4.0	22(10.5)	9(9.9)			22(10.5)	9(10.7)	0(0)	
Median (Range)	1.40 (0.4–5.4)	1.40 (0.4–5.8)	------	§0.564	1.40 (0.4–5.4)	1.40 (0.4–5.8)	2.40 (0.6–3.1)	§0.603
**Leucocyte (× 10**^**9**^**/L)**								
< 4.0	44(21.1)	17(18.7)	------	#0.614	44(21.1)	16(19.0)	1(14.3)	#0.894
4.0–10.0	95(45.5)	47(51.6)			95(45.5)	43(51.2)	4(57.1)	
> 10.0	70(33.5)	27(29.7)			70(33.5)	25(29.8)	2(28.6)	
Median (Range)	7.7 (1.9–16.2)	7.8 (1.8–15.2)	------	^0.872	7.70 (1.9–16.2)	7.75 (1.8–15.2)	9.40 (2.8–12.2)	^0.935
**Platelets**								
≤ 100	28(13.4)	16(17.6)	0.73 (0.37–1.42)	&0.376	28(13.4)	15(17.9)	1(14.3)	&0.621
> 100	181(86.6)	75(82.4)			181(86.6)	69(82.1)	6(85.7)	
Median (Range)	245.0 (71–420)	241.0 (76–402)	------	§0.572	245.0 (71–420)	240.0 (76–402)	259.0 (86–320)	§0.724
**CRP level (mg/L)**								
≤ 5	43(20.6)	7(7.7)	3.11 (1.34–7.21)	&0.006*	43(20.6)	7(8.3)	0(0)	&0.019*
> 5	166(79.4)	84(92.3)			166(79.4)	77(91.7)	7(100)	
Median (Range)	48.0 (4–768)	48.0 (4–768)	------	§0.823	48.0 (4–768)	48.0 (4–768)	48.0 (12–768)	§0.567
**Ferritin level (μg/L)**								
≤ 300	77(36.8)	23(25.3)	1.73 (1.00–2.99)	&0.062	77(36.8)	23(27.4)	0(0)	&0.050*
> 300	132(63.2)	68(74.7)			132(63.2)	61(72.6)	7(100)	
Median (Range)	387.0 (12–2500)	500.0 (10–2360)	------	§0.124	387.0 (12–2500)	471.0 (10–2360)	932.0 (369–1880)	§0.028*
**D-dimers level (μg/L)**								
< 0.5	90(43.1)	30(33.0)	------	#0.104	90(43.1)	29(34.5)	1(14.3)	#0.009*
0.5–1.0	65(31.1)	27(29.7)			65(31.1)	27(32.1)	0(0)	
> 1.0	54(25.8)	34(37.4)			54(25.8)	28(33.3)	6(85.7)	
Median (Range)	0.60 (0.1–3.3)	0.80 (0.1–3.1)	------	§0.017*	0.60 (0.1–3.3)	0.70 (0.1–3.1)	2.0 (0.4–2.8)	§0.002*
**Imaging**								
**CT findings**								
Normal	23(11.0)	4(4.4)	0.37 (0.13–1.11)	&0.079	23(11.0)	4(4.8)	0(0)	&0.169
Bilateral GGO	142(67.9)	63(69.2)	1.06 (0.62–1.81)	&0.893	142(67.9)	58(69.0)	5(71.4)	&0.968
Pneumonic consolidation	69(33.0)	38(41.8)	1.46 (0.88–2.41)	&0.152	69(33.0)	35(41.7)	3(42.9)	&0.347

^^^Independent t-test

^#^Chi square test

^§^Mann Whitney test

^&^Fisher's Exact test. *p* values represent statistical significance < 0.05

^*^Significant. *OR* Odds ratio. *CI* confidence interval. *CRP* C-reactive protein, *CT* Computed Tomography, *GGO* Ground-glass opacity, *L* liter, *mL* millileter, *mg/L* milligram/Liter, *μg/L* microgram/Liter

### Treatment and outcomes according to CXCL12 rs2839693 genotypes

As indicated in Table 8, a significant difference between genotypes regarding clexane as a treatment was detected (*P* value 0.022). Clexane was more prevalent in the TT genotype than the CC and CT genotype. The number of patients without any demand for oxygen therapy was significantly different between the genotypes (*P* value < 0.001) where it was more prevalent in the CC genotype compared with the combined CT + CC and the TT genotypes. Nasal cannula and invasive mechanical ventilation were more prevalent in the TT and the combined CT + TT genotypes compared with the combined CC, CT and the CC genotypes, respectively, (*P* value < 0.001 and 0.002 for nasal cannula, 0.025 and 0.031 for invasive mechanical ventilation). Mask oxygen was used more prevalently in patients of the CT + TT genotypes than those of the CC genotype (*P* value 0.044).

**Table 8:** Comparison between genotypes (CXCL12 rs2839693) according to treatment and outcomes

	**CXCL12 rs2839693**							
	**CC** **(*n* = 209)**	**CT + TT** **(*n* = 91)**	**OR (95%CI)**	*P* value*	**CC** **(*n* = 209)**	**CT** **(*n* = 84)**	**TT** **(*n* = 7)**	*P* value*
	**No. (%)**	**No. (%)**			**No. (%)**	**No.(%)**	**No.(%)**	
Antibiotic	203(97.1)	85(93.4)	0.42(0.13–1.34)	&0.196	203(97.1)	78(92.9)	7(100)	&0.207
Antifungal	12(5.7)	6(6.6)	1.16(0.42–3.19)	&0.794	12(5.7)	6(7.1)	0(0)	&0.717
Antiviral	155(74.2)	77(84.6)	1.92(1.00–3.66)	&0.052	155(74.2)	71(84.5)	6(85.7)	&0.138
Glucocorticoids	116(55.5)	60(65.9)	1.55(0.93–2.59)	0.099	116(55.5)	55(65.5)	5(71.4)	&0.230
Clexane	57(27.3)	35(38.5)	1.67(0.99–2.81)	&0.058	57(27.3)	30(35.7)	5(71.4)	&0.022*
**Oxygen therapy**								
None	64(30.6)	9(9.9)	0.25(0.12–0.53)	& < 0.001*	64(30.6)	9(10.7)	0(0)	& < 0.001*
Nasal cannula	43(20.6)	35(38.5)	2.41(1.41–4.14)	&0.002*	43(20.6)	29(34.5)	6(85.7)	& < 0.001*
Mask oxygen	96(45.9)	54(59.3)	1.72(1.04–2.83)	&0.044*	96(45.9)	50(59.5)	4(57.1)	&0.102
Invasive mechanical ventilation	8(3.8)	10(11.0)	3.10(1.18–8.14)	&0.031*	8(3.8)	10(11.9)	0(0)	&0.025*
**Outcomes**								
Home management	65(31.1)	13(14.3)	0.37(0.19–0.71)	&0.002*	65(31.1)	13(15.5)	0(0)	&0.006*
Hospitalization without ICU	61(29.2)	17(18.70	0.56(0.30–1.02)	&0.063	61(29.2)	17(20.2)	0(0)	&0.082
ICU	83(39.7)	61(67.0)	3.09(1.84–5.18)	& < 0.001*	83(39.7)	54(64.3)	7(100)	& < 0.001*
Death	21(10.0)	18(19.8)	2.21(1.11–4.38)	&0.026*	21(10.0)	13(15.5)	5(71.4)	& < 0.001*
**Duration of ICU stay (days)**								
Median( Range)	8.00(1–13)	7.00(1–13)	------	§0.284	8.00(1–13)	6.50(1–13)	9.00(1–12)	§0.159
**Duration of in-hospital stay (days)**								
Median(Range)	7.50(1–15)	7.00(1–15)	------	§0.856	7.50(1–15)	7.00(1–15)	12.00(6–15)	§0.035*
**Duration of recovery (days)**								
Median (Range)	18.00(2–48)	15.00(2–48)	------	§0.703	18.00(2–48)	15.00(2–48)	23.50(2–44)	§0.930

^§^Mann Whitney test

^&^Fisher's Exact test. *p* values represent statistical significance < 0.05

^*^Significant. *OR* Odds ratio. *CI* confidence interval. *ICU* Intensive care unit

The number of patients needed home management was significantly higher in the CC genotype compared with the combined CT + TT and the TT genotypes (*P* value 0.002 and 0.006 respectively). The need for ICU stay was significantly higher in patients with the combined CT + TT and the TT genotype compared with CC and CC, CT, respectively, (*P* value < 0.001 and < 0.001 respectively). Regarding mortality rates, our results revealed that they were significantly higher in the combined CT + TT and the TT genotype compared with the CC and the CC, CT, respectively (*P* value 0.026 and < 0.001 respectively).Patients with the TT genotype had a longer duration of in-hospital stay than patients with the CC genotype (median, 12.0 days, as compared with 7.5 days; *P* value 0.035).

## Discussion

Numerous investigations have been carried out since the COVID-19 tragedy first emerged in order to comprehend the disease's mechanics and determine the cause of the variation in symptoms across patients. Because of their importance in the cytokine storm and the onset of ARDS, chemokines and their receptors were among the most significant elements that were researched in this context. Therefore, it may be possible to forecast the results of COVID-19 by fully comprehending the signature of chemokines and their receptors. Therefore, we proposed that CXCL12 could influence the results and severity of COVID-19.

Our present research examined 156 mild or moderate and 144 severe or critical COVID-19 patients at Assiut University Quarantine Hospital from a cross-sectional perspective view.

Most of our participants were vaccinated (69.3% compared with 30.7% non-vaccinated patients) and non-smokers (60.7% compared with 39.3% smokers). Hypertension and DM were the most common co-morbid diseases found in 35.7% and 26.0% patients respectively; while renal disease, heart disease, and thyroid disease were less frequent. These findings agree with a study by Petrakis et al., who reported that the most frequent comorbid diseases were hypertension in 38.4% patients and DM in 20.9% patients [19].

Additionally, regarding clinical manifestations of the studied groups, The principal COVID-19's clinical complaints were fatigue (55.0%), dry cough (53.7%), and dyspnea (48.0%).These findings are similar to those by Rodriguez-Morales et al., who reported that the principal COVID-19's clinical complaints were cough(57.6), and dyspnea (45.6); in contrast to our results fatigue (29.4%) [20].

Regarding laboratory assessment, lymphocytes values in most cases 185 (61.7%) were typically within the normal values between (1.0–4.0 × 10^9^/L), while lymphopenia was observed in 84 (28.0%) patient. Ferritin and CRP were elevated in most cases 200 (66.7%) and 250 (83.3%), respectively. Moreover, D-dimers were elevated in 180 (60.0%) of our cases. Our results in agree with Kadhim et al., and Smail et al., who observed lymphopenia, elevated CRP, ferritin, and D-dimers among COVID-19 patients [21, 22].

Bilateral GGO was the most frequent CT abnormality 205 (68.3%). Pneumonic consolidation was another prevalent CT characteristic in COVID-19 patients 107 (35.7%). These findings are similar to a meta-analysis research conducted on 13 previous studies and concluded that GGO was the principal CT findings followed by consolidation in COVID-19 patients [23].

Most of our participants received antibiotic as a treatment 288 (96.0%) patients; while 323 (77.3%) and 176 (58.7) patients received antiviral and Glucocorticoids, respectively. Oxygen therapy was needed in 227 (75.7%) patients: nasal cannula in 78 (26.0%), Mask oxygen in 150 (50.0%), and Invasive mechanical ventilation in 18 (6.0%). A meta-analysis study by Langford et al., on the use of antibiotic therapy on COVID-19 found that the frequency of antibiotic therapy was 74.6% [24].

The average length of hospitalization was 7.0 days. These findings agree with Alwafi et al., who indicated that the average length of hospitalization was 6.0 days [25]. While the median duration of ICU stay was 7.0 days. In contrast to our results, López-Cheda et al., indicated that the median duration of ICU stay was 14.0 days [26].

The median duration of recovery was 15.0 days. Our data agree with SeyedAlinaghi et al., who found that the median length of recovery was 13.5 days [27]. Regarding mortality rate, our data revealed that 39 (13.0%) died. These results agree with data collected from 20 regions in Italy by Immovilli et al., and revealed that the mortality rate ranged between 3.1% and 16.7% [28].

The frequency of the TT genotype and the T allele of CXCL12 rs2839693 was significantly different between mild or moderate and severe or critical patients. The frequency of the TT genotype and the T allele was higher in the severe or critical group than in the mild or moderate which may indicate the role of CXCL12 rs2839693 in the pathogenesis and severity of COVID-19. Patients with the TT genotype or the T allele at increased risk developed severe or critical rather than mild or moderate one. Our result agree with Mohamed et al., who reported no differences in the frequency of the TT genotype or the T allele of CXCL12 rs2839693 among ITP patients and controls, and that there is no correlation between this SNP and disease severity [13].

Normal CXCL12 is the only ligand that could bind to CXCR4 receptor. SARS-CoV-2 acts as a competitor for CXCL12 on CXCR4 receptor. The reason for why patients with TT genotype possess a severe form of the disease may be due to the impact of the polymorphisms on CXCL12 that makes it works dysfunctionaly. So, it keeps the way clear for SARS-CoV-2 to bind CXCR4 as a co-receptor leading to increase viral load and disease severity.

CXCL12 has a function in inflammation resolution, for example, by increasing angiogenesis and tissue repair. Prolonged CXCL12 activity may not only improve leukocyte chemotaxis, which is advantageous, but it may also worsen the chronic inflammation identified in COVID-19 [29]. CXCL12 expression rises with autoimmune disorders. CXCL12 levels are raised in individuals with a range of inflammatory illnesses, indicating that CXCL12 plays a role in autoimmunity [30].

The expression of CXCL12 is elevated in the Fibroblasts from severe COVID-19 patients which may help to attract immune cells with the CXCR4 receptor, such as macrophages, T cells, and NK cells [31]. Previous studies have found higher levels of CXCL12 in the blood of severe COVID-19 patients hospitalized in critical care units when compared to hospitalized patients with mild to moderate illness and/or healthy controls [32].

CXCL12 gene polymorphisms might influence the regulation of ACE2, the receptor used by the SARS-CoV-2 virus to enter human cells. Altered ACE2 expression or function could impact viral entry and disease progression. Patients with severe COVID-19 have much higher levels of ACE2 expression, which allows more viral invasiveness [33].

Gene polymorphisms in CXCL12 might lead to differences in immune cell recruitment and activation, which could affect the immune response to the virus and the development of severe COVID-19. Effective hematopoiesis, homing of T and memory B cells to lymph nodes, and monocyte recruitment depend on CXCL12, the CXCR4 ligand. Several viruses employ the inhibition of this axis to reduce the number of circulating immune cells and raise their own proliferative rate [32].

CXCL12 polymorphisms could potentially impact the ability to clear the virus from the body, affecting the duration and severity of COVID-19. Cellular clearance of the virus depends on the production of virus-specific antibodies, which block the entry of free virions into uninfected cells, opsonize the virus for inactivation by complementing proteins or elimination by phagocytic immune cells that contain CXCL12 receptor like macrophages and neutrophils. They also inactivate or start the killing of infected cells by activating the complement cascade and through antibody-mediated cytotoxicity processes, essential for [34].

Elimination of SARS-CoV-2 results in either an earlier time of recovery or a decrease in the severity of disease. According to Zheng, F. et al., the removal of viral shedding after viral clearance in patients would also aid to lessen viral transmission [35].

In agreement of our data Wang et al., found that patients with CXCL12 rs2839693 were associated with increased susceptibility to sepsis [36]. Zhang et al., conducted a study on 597 patients with coronary artery disease which revealed that CXCL12 rs2839693 TT was associated with increased risk to coronary artery disease in men [12].

The multivariate logistic regression analysis of the genotypes against clinical COVID19 revealed that the combined genotype CT + TT acts as independent predictor for the severe or critical clinical condition of COVID 19.

According to CXCL12 rs2839693 genotypes, our results revealed a non-significant difference among genotypes regarding age, gender, vaccination, and Co-morbid diseases. On the other side, regarding symptoms, our findings showed significant associations of genotypic distributions with dyspnea, vomiting, and dysarthria of COVID-19 patients. Dyspnea was more prevalent in the combined CT + TT and the TT genotypes compared with the CC and the CT, CC genotypes, respectively. Vomiting and dysarthria were significantly prevalent in the TT genotypes compared with the CT, CC genotypes. Regarding clinical course of the disease, we found that severe or critical illness associated with the combined CT + TT and the TT genotypes was more prevalent in the combined CT + TT and the TT genotypes compared with the CC and the CT, CC genotypes respectively.

According to laboratory evaluation, our results showed significant differences between genotypes as regard to CRP, D-dimers, and ferritin. CRP and D-dimers are significantly elevated in the combined CT + TT and the TT compared with the CC and the CC, CT genotypes; while ferritin was significantly elevated in the TT compared with the CC, CT genotypes. Additionally, no significant differences were found between the 3 genotypes as regarding lymphocyte count, leucocyte count, and CT findings.

Regarding treatment, a significant difference between genotypes as regarding clexane as a treatment was detected; Clexane was more prevalent in the TT genotype than the CC and the CT genotype. The number of patients without any demand for oxygen therapy was significantly different between the genotypes where it was more prevalent in the CC genotype compared with the combined CT + CC and the TT genotypes. Nasal cannula and invasive mechanical ventilation were more prevalent in the TT and the combined CT + TT genotypes compared with the combined CC, CT and the CC genotypes, respectively. Mask oxygen was used more prevalently in the patients of the CT + TT genotypes than those of the CC genotype.

The number of patients needed home management was significantly higher in the CC genotype compared with the combined CT + TT and the TT genotype. The need for ICU stay was significantly higher in patients with the combined CT + TT and the TT genotype compared with the CC and the CC, CT, respectively. Regarding the mortality rates, our results revealed that they were significantly higher in the combined CT + TT and the TT genotype compared with the CC and the CC, CT, respectively. Patients with the TT genotype had a longer duration of in-hospital stay than patients with the CC genotype.

Our findings may provide new insights on understanding the different factors affecting disease severity and mechanisms of cytokine storm syndrome which could affect COVID-19 outcomes and treatment strategies.

There are two limitations that we encountered while conducting this study. First, the number of patients was limited due to the difficulty in obtaining samples from critical cases, cases without symptoms, or cases that had recovered from the disease. Secondly, some cases were removed from the study due to negative smears or some comorbidities that are not suitable for the study.

### Recommendations

The present study recommends studying other SNPs in the CXCL12 gene and their relationship with COVID-19 pathogenicity and severity. It also recommends expanding the study patient population to better understand the effect of CXCL12 gene variations on COVID-19 patients.

## Conclusion

Our data indicated the signature of CXCL12 rs2839693 in the pathogenesis and severity of COVID-19. Patients with the TT genotype or the T allele at increased risk developed severe or critical rather than mild or moderate disease. Also, patients' admission circumstances, such as vaccination, comorbidities, and symptoms may indicate disease severity. These variables require additional exploration and should be taken into account for risk categorization.

## Data Availability

The datasets generated and/or analysed during the current study are available at biosample depository, SubmissionID: SUB13604667 https:// ncbi.nlm.nih.gov /subs/biosample/SUB13604667.

## Abbreviations

C: Degree Celsius
ACE 2: Angiotensin-converting enzyme II
ARDS: Acute respiratory distress syndrome
BCG: Bacille Calmette-Guérin
COPD: Chronic obstructive pulmonary disease
COVID-19: Coronavirus disease 2019
CT: Computed tomography
ICU: Intensive care unit
IFN-γ: Interferon-gamma
PCR: L: liter
mL: Millileter, mg/L: milligram/Liter, μg/L: microgram/Liter, Polymerase chain reaction
RT-PCR: Reverse transcription polymerase chain reaction, SARS-CoV-2: Severe acute respiratory syndrome coronavirus 2
SpO2: Oxygen saturation
TMPRSS2: Transmembrane protease serine 2
TNF-α: Tumor necrosis factor-alpha
VOCs: Variants of concern
WHO: World Health Organization
